# An FPGA-based system for generalised electron devices testing

**DOI:** 10.1038/s41598-022-18100-3

**Published:** 2022-08-17

**Authors:** Patrick Foster, Jinqi Huang, Alex Serb, Spyros Stathopoulos, Christos Papavassiliou, Themis Prodromakis

**Affiliations:** 1 Centre for Electronics Frontiers, Zepler Institiute, University of Southampton, Southampton, UK; 2 School of Engineering, University of Edinburgh, Edinburgh, UK; 3 Department of Electrical and Electronic Engineering, Imperial College London, London, UK; 4 ArC Instruments, Southampton, UK

**Keywords:** Electrical and electronic engineering, Characterization and analytical techniques, Characterization and analytical techniques

## Abstract

Electronic systems are becoming more and more ubiquitous as our world digitises. Simultaneously, even basic components are experiencing a wave of improvements with new transistors, memristors, voltage/current references, data converters, etc, being designed every year by hundreds of R &D groups world-wide. To date, the workhorse for testing all these designs has been a suite of lab instruments including oscilloscopes and signal generators, to mention the most popular. However, as components become more complex and pin numbers soar, the need for more parallel and versatile testing tools also becomes more pressing. In this work, we describe and benchmark an FPGA system developed that addresses this need. This general purpose testing system features a 64-channel source-meter unit, and $2\times$ banks of 32 digital pins for digital I/O. We demonstrate that this bench-top system can obtain ${170}\,\hbox {pA}$ current noise floor, ${40}\,\hbox {ns}$ pulse delivery at $\pm {13.5}\,\hbox {V}$ and ${12}\,\hbox {mA}$ maximum current drive/channel. We then showcase the instrument’s use in performing a selection of three characteristic measurement tasks: (a) current–voltage characterisation of a diode and a transistor, (b) fully parallel read-out of a memristor crossbar array and (c) an integral non-linearity test on a DAC. This work introduces a down-scaled electronics laboratory packaged in a single instrument which provides a shift towards more affordable, reliable, compact and multi-functional instrumentation for emerging electronic technologies.

**Figure 1:**
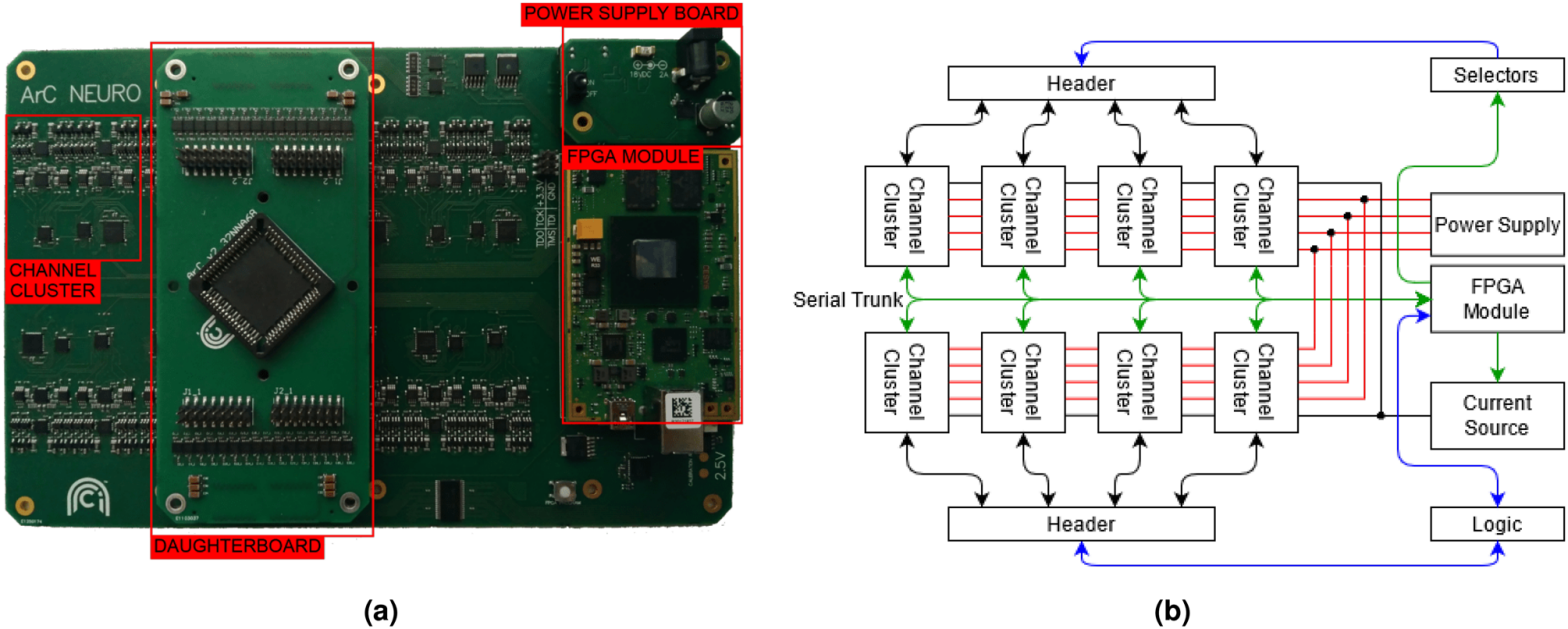
Overview of instrument. (**a**) Picture of fully assembled system PCBs, including base board, device-under-test interfacing daughterboard, FPGA dev board and power supply board. (**b**) High-level block diagram of system architecture illustrating parallelism and modularity of the system. Analogue connections are shown in black, serial connections are shown in green, parallel connections are shown in blue, and power supply connections are shown in red.

## Introduction

Progress of electronic technologies has relied on a solid foundation of instrumentation tools ranging from single components, such as instrumentation amplifiers^1^, and high-end data converters^2^, to small-size printed circuit board (PCB) instruments for generalised parameter measurements^3,4^, benchtop instruments such as oscilloscopes and signal generators. These instruments have defined both the limits of what can be measured and tested, and play a significant role in determining the productivity of laboratories around the world. In fact, it is particularly the latter that has led to the development of specialist instrumentation such as lock-in amplifiers^5^ and spectrum analysers^6^.

Over time, both the variety and complexity of circuits being developed and requiring testing is increasing. As an example let us consider the story of instrumentation for the emerging memory devices (including memristors) community^7^. These devices act as electrically tuneable resistors and hence require analogue instrumentation for their characterisation with typical tests being current-voltage sweeps, incremental-step pulse programming^8^. Moreover, Resistive Random Access Memory (RRAM) memristive devices are very frequently used as crossbar arrays for performing dot products^9^. This need has led to the development of lightweight instrumentation emphasising parallelism and speed of data acquisition over raw accuracy^10–12^. This, in turn, has implied significant circuit design effort to mitigate effects related to sneak paths^13^, which were shown to lead to potentially catastrophically undermine read-out accuracy via a variety of imperfection mechanisms^14,15^. Nonetheless, these array-level instruments were soon superseded by increasing complexity in RRAM crossbar arrays with the popularisation of the so-called ‘1T1R’ approach^16^, where each RRAM device is paired with a ‘selector transistor’, thus now requiring a new set of control terminals for the gates of the transistors (as shown later in Fig. 10). In parallel, advances in RRAM technology have led to memristor cells capable of ever finer gradations of their resistive states^17^, which has been pushing the accuracy requirements of instrumentation upwards.

The exemplar story of RRAM instrumentation illustrates the trend towards higher ‘device under test’ and ‘circuit under test’ complexity, with numerous other examples easy to draw from precision amplifiers with 20 pins^18^ to multi-channel switches and data converters^2^, micro-controllers^19^ etc. In response to this trend, several designs have been dedicated to the testing systems for general electronic devices or specific devices such as RRAM. Wust, D. et al.^20^ developed a field programmable gate array (FPGA) based memristor prototyping environment, but with a maximum theoretical resolution of ${740}\,\hbox {pA}$, this system cannot deliver more detailed characterisation tasks. Berdan, R. et al.^10^ implemented a microcontroller-based advance testing system for memristor devices, but the parallelism is limited. Wang, Y. et al.^21^ presented a high-speed driving system for phase change memory devices, with the narrowest pulse width of 500ns. However, this work only has a driver side. Other works such as Merced-Grafals, E. et al.^22^ applied commercially available device analysers, which have limited channel numbers as well as parallelism. In continuation of our previous work in the field of RRAM instrumentation, we have developed a new instrument with the purpose of being highly parallel, competitively accurate to heavier bench-top instruments, easily transportable, and flexible enough to test circuits with up to a maximum of 128 pins with an array of analogue and digital source and metering capabilities.

In this paper, we present the scientific contributions resulting from the development of this new instrument, namely: (i) the design and implementation of a general purpose, 64-channel fully-parallel analogue source-metre unit (SMU) with specialist circuitry introduced to allow (a) current-mode biasing and (b) high-speed pulsing capability (tackled in section "System implementation") and (ii) the benchmarking of the SMU’s performance in terms of accuracy, noise floor and pulsing characteristics (section "Experimental results"). Furthermore, we illustrate how the instrument can be used flexibly via presenting three practical examples: characterising a transistor, interfacing a RRAM crossbar array and testing the differential non-linearity (DNL) of a data converter (in section "Application examples") and conclude the paper (section "Discussion and conclusions)" by discussing the opportunities arising.

## System implementation

The system we have developed is shown in Fig. 1b. It comprises a 64-channel, fully parallel SMU array and $2\times$ banks of 32 digital pins. The instrument also features a shared current source. The entire system is coordinated by an FPGA EFM-03 development board with Xilinx XC7A200T-2FBG676I chip and is controlled by a PC. The computer control of the tool consists of a low-level Rust library which exposes a Python Application Programming Interface (API). By leveraging the Python API a Qt-based Graphical User Interface has been built which is focused on crossbar-level testing (see Data Availability Statement for links to the repositories).

The system has been engineered to provide high-throughput, parallel testing at high-levels of accuracy. The assembled instrument is shown in Fig. 1a, with the standard interfacing daughter-board (for connecting to PLCC68 packages). The power supply daughter-board and FPGA development board can also be seen.

### Subsystem overview

**Figure 2:**
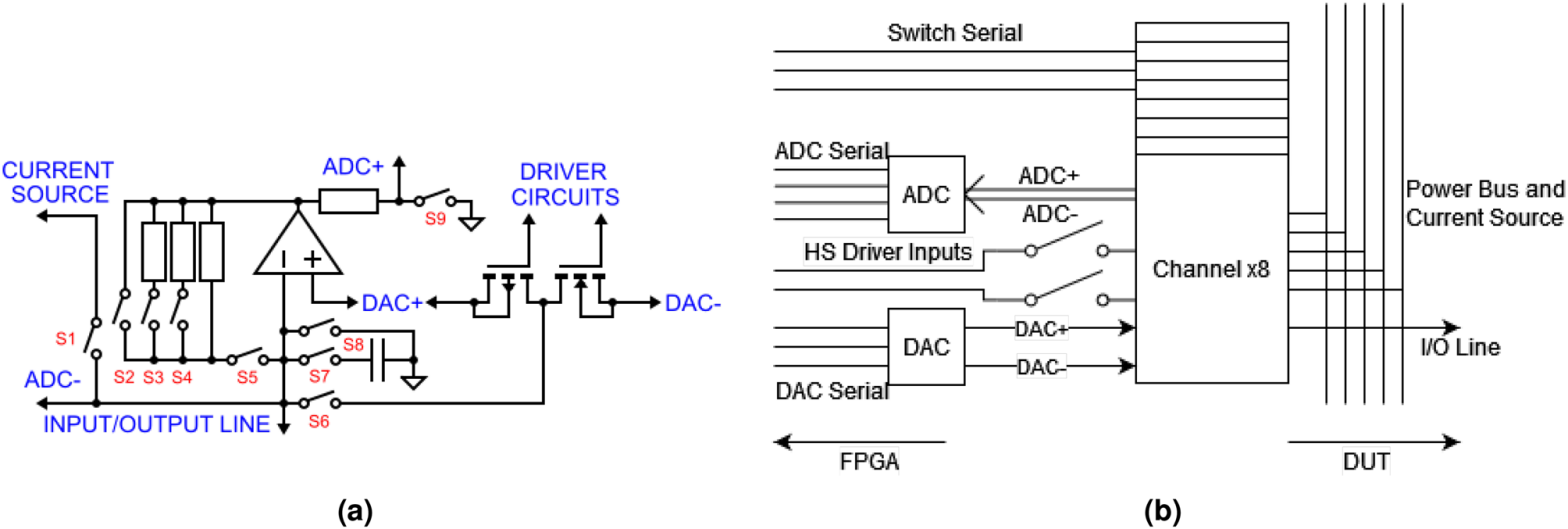
(**a**) is a schematic of the channel architecture. Significant wires are labelled in blue. Analogue switches are labelled in red. (**b**) is a schematic of the structure of the channel cluster.

The main subsystem of the board is the SMU channel. It consists of: (a) a programmable gain trans-impedance amplifier (TIA), (b) an independent pulse generator used for high-speed pulsing and (c) a switch which allows the channel to access the current source, as shown in Fig. 2a. Data converter terminals are connected as shown in Fig. 2a to provide biasing with digital to analogue converters (DACs). This allows the channel to act as a tuneable source, or to read voltages with differential analogue to digital converters (ADCs) at selected nodes for measurement.

The TIA structure is designed to act as either a source or a meter for the Input/Output (I/O) node. In voltage source mode, the TIA feedback path is connected as a short circuit with S2 and S5, allowing the TIA to act as a unity gain buffer for the DAC+ output. The time required for this operation is determined by the slew rate of the DAC+ reference, at ${0.4}\,\hbox {V}/{}\,\upmu \hbox {s}$. To meter current the TIA is set to the appropriate gain with S3-5, causing a voltage drop across the feedback path proportional to the current required to bring the node to the same voltage as DAC+. With the S9 switch open, this voltage is applied to the input of the ADC, regardless of the set voltage of the DAC+ reference. It takes around ${900}\,\upmu \hbox {s}$ for the TIA to settle in its most sensitive range and ${320}\,\upmu \hbox {s}$ to take a 32 sample average, for around ${1.2}\,\hbox {ms}$ total delay. To measure voltage the S9 switch can be closed, referencing the ADC against ground rather than the TIA output. This can take either ${10}\,\upmu \hbox {s}$ or ${320}\,\upmu \hbox {s}$, depending on if averaging is used.

The amplifier selected for this design was chosen for its low quiescent current and input bias current, but this comes at the cost of frequency response, with a gain-bandwidth product of only ${2.5}\,\hbox {MHz}$. While this limits the rate at which the TIA can settle, the time required for a current reading is only slightly longer than previous work^23^. In spite of this, the ability to read an entire line of an array at once reduces the time required to read a 32 by 32 array by a factor of 20 to 80, depending on the state of the devices in the array. The analogue switches were chosen for a balance of low on resistance and charge injection, with ${9.5}\,{\Omega }$ and ${4}\,\hbox {pC}$ respectively.

The high-speed pulse driver is implemented with a complementary MOSFET pair which can drive the output line to the voltage of either of two DAC channels. This connectivity allows for variable pulse amplitude and enables high-speed by keeping the path between charge supplier and I/O line very simple and low impedance. Bi-phasic pulses can be constructed across 2-terminal devices by having two channels swing between $V_+>0$ and 0 and $V_-<0$ and 0 respectively.

The switch S1 connects the I/O line to the shared current source, to permit current biasing. Current biasing can also be achieved through the TIA by using successive approximation, if parallel operation is required, but precise current control requires a dedicated circuit which is too large to be included in the channel. As a result of sharing, a more complex dedicated current source could be designed to source or sink sub-*nA* currents, permitting current biasing of $\hbox {G}\Omega$ scale resistive devices. The current source circuit also contains a precision voltage reference, which can be connected to any channel to calibrate the ADC. At a higher level, the individual SMU channels are grouped into clusters of eight (Fig. 2b). This allows each cluster to share one 8 channel 18-bit ADC and one 16 channel 16-bit DAC. To further reduce the number of control pins per cluster, the high speed driver control signals are unified into a single pair through an array of analogue switches. The analogue switch IC used in this design has an integrated serial FIFO register, allowing the switches of all the channels in a cluster to be controlled in single serial daisy-chain. The switch, ADC, and DAC serial lines from each cluster are grouped together into a bus that runs down the centre of the board, called the serial trunk. The cluster is physically arranged so that all control signals are on one side, with the measurement lines on the other and the supply rails running perpendicular on a different layer. Each cluster also shares the same control signals for the high-speed drivers. Channels in separate clusters can produce asynchronous pulses, but channels in the same cluster cannot.

The next subsystem is the digital pin banks. The first bank of 32 channels (the ‘selector’ bank) is an output-only set that is intended to drive transistor gates. This was developed to address the needs of selector transistors in RRAM arrays^24^. As a result, the HI and LO voltages can be set arbitrarily, but they are common for the entire bank. Furthermore, both drive strength and speed are relatively low. The second bank (the ‘arbitrary logic bank’) is a more conventional full digital I/O system, which is referenced exclusively to GND. It is intended to drive digital pins on test chips or read from them.

### Digital interface hierarchy

**Figure 3:**
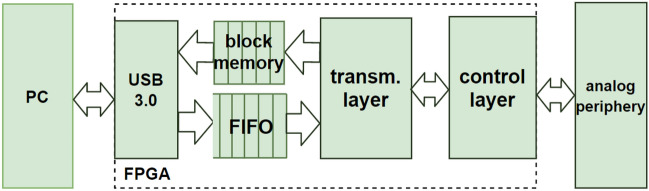
Digital interface hierarchy. The speed of internal data bus is 3.2 Gbps.

Figure 3 illustrates the concept diagram of the digital interface, which bridges the gap between the PC-level software and the analogue circuitry of the PCB board. The basic structure of the digital interface contains a USB 3.0 IP core, a first-in-first-out (FIFO) buffer, block memory, a transmission layer and a control layer. The instruction set has been designed for translating a relatively small set of high-level operations into “board language”. These are: select channels, emit pulse, read from channel(s) as well as set current (for the shared current source) and a few more specialised commands. In hardware, this translates to configuring the high-speed pulse drives, DACs, ADCs, switches and digital pins. All advanced functions can be performed through a combination of the basic set of commands. The transmission layer performs the translation from PC-level instructions to PCB-level and the control layer executes the latter.

As an example, a basic write operation needs commands to configure the high-speed pulse driver and SMU channel switches (see Fig. 2a). Information such as voltage pulse amplitude, pulse width and target devices will be processed and converted on the PC. Then, the FPGA will receive the commands through USB3.0, configure the target channel and then trigger the pulse. Information flows in the opposite direction in a basic read operation. Commands for DACs and ADCs are sent to configure the bias voltage and start voltage readout in the selected channels. The measurement results are temporarily stored in the on-chip memory of the FPGA waiting for the PC to be ready to process it. To match the PC-side and FPGA-side speeds of transmission and processing, a FIFO buffers the PC-to-FPGA downlink and a block memory buffers the uplink. The FIFO can currently fit just one instruction package, but will eventually be upgraded to 32+ instructions.

All IPs inside the FPGA are linked through an Advanced eXtensible Interface (AXI). AXI is a universal high-speed high-performance interface, typically used in microcontroller systems^25^. The burst-based property of AXI and 100MHz FPGA system clock allows internal data transmission rates of up to 3.2 Gbps . The third-party USB3.0 IP^26^ we used also generated 100MHz clock for USB controller chip CYUSB3014^27^, giving the same maximum 3.2Gbps data rate for communication via usb.

## Experimental results

Benchmarking the instrument involved performing a set of experiments to determine the noise floor of voltage and current read operations, the read-out accuracy of test resistances, the pulse characteristics obtained at the when using the write functions of the system and some basic data on the functionality of the digital terminals.

### Noise floor

**Figure 4:**
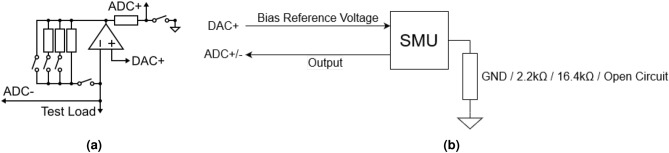
(**a**) functional schematic of the channel extracted from Fig. 2a. (**b**) high-level schematic indicating the loading configuration used in the test.

**Figure 5:**
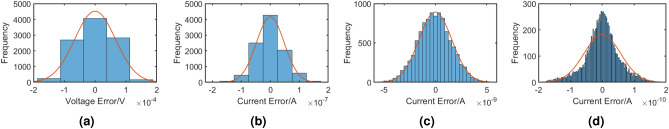
Histograms showing noise characteristics of the various modes of measurement. All histograms have one bin per ADC code with widths of ${78.1}\,\upmu \hbox {V}$, ${47.6}\,\hbox {nA}$, ${355}\,\hbox {pA}$, and ${2.60}\,\hbox {pA}$ respectively. (**a**) 10k point histogram of a read-out voltage error test (V=GND), overlaid with Gaussian distribution estimate. We obtain $\sigma ={66}\,{\mu \hbox {V}}$. (**b**–**d**) 10k point histograms of current read-out tests, overlaid with Gaussian distribution estimates. (**b**) ${820}\,{\Omega }$ TIA range yields $\sigma ={48}\,\hbox {nA}$. (**c**) the ${110}\,{\hbox {k}\Omega }$ TIA range yields $\sigma ={1.6}\,\hbox {nA}$. (**d**) the ${15}\,\hbox {M}\Omega$ TIA range yields $\sigma ={57}\,\hbox {pA}$.

To assess the noise floor of 32 sample average voltage readings, we grounded a channel (Fig. 4) and collected 10k voltage readings as shown in Fig. 5a. The voltage readings mostly spanned across three consecutive ADC codes. Using a Gaussian noise model we estimated standard deviation (s.d.) of ${66}\,{\upmu \hbox {V}}$, although this may not be accurate due to the variance being of similar scale to the quantisation error.

To assess the noise floor of 32 sample average current readings, we configured a channel as a TIA with a reference of ${-0.5}\,\hbox {V}$, thus subjecting the test load to that voltage. We then connected different resistors between the input node and ground to produce a bias current that forces the channel to automatically select a specific range (Fig. 4), and then collected 10k readings in each range, as shown in Fig. 5. The uncertainty in the instrument’s readings was thus obtained. For the ${820}\,{\Omega }$ range, we connected a ${2.2}\,\hbox {k}\Omega$ resistor. As with the voltage readings, in this range the results mostly spanned across just three consecutive ADC codes (Fig. 5b). This suggests to us that the noise in this range is dominated by the ADC noise and quantisation error. Using a Gaussian noise model we estimated s.d. of ${48}\,\hbox {nA}$. The test was repeated with a ${16.4}\,\hbox {k}\Omega$ resistor, targeting the ${110}\,\hbox {k}\Omega$ TIA gain range (Fig. 5c). The distribution was approximately Gaussian, with an s.d. of ${1.6}\,\hbox {nA}$, or roughly 5 LSB. To test the ${15}\,\hbox {M}\Omega$ TIA range, we left the TIA input open circuit and obtained s.d. of $\sigma ={57}\,\hbox {pA}$, or roughly 22 LSB. The error distribution in this range did not display the Gaussian distribution obtained in tests of other ranges. Experimentation showed that the extended tail of the distribution was a result of mains interference: During each test, the wires used to connect resistors for preceding tests was left in place; removing these (thereby reducing the length of floating input line) resulted in reduced uncertainty. The input lines of the channel act as an antenna, collecting energy emitted from nearby mains wiring. All results presented here for the lowest current range represent the test wires removed to minimise antenna effects. This could likely be eliminated by operating the instrument inside an anechoic chamber, adding load capacitance or other good measurement techniques applied independent of the instrument.

### Read-out accuracy

**Figure 6:**
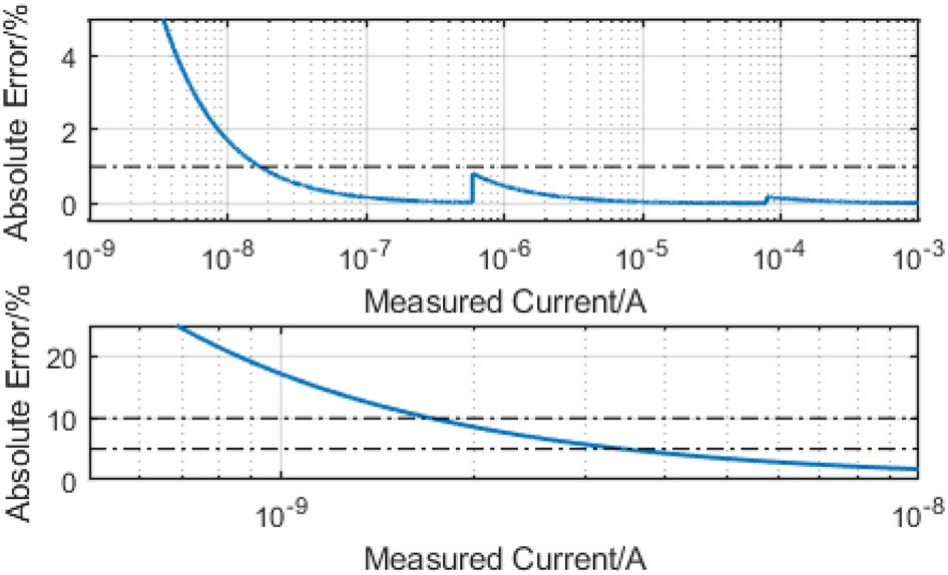
Graph showing predicted absolute error based on $3\sigma$ current noise error.

We calculated the ‘reasonable worst-case’ proportional current reading error across the designed operating range of the instrument by assuming a measurement error of $3\sigma$ (Fig. 6). Current measurements of more than ${16}\,\hbox {nA}$ can be made with 1% accuracy, at a sampling rate of ${833}\,\hbox {Hz}$. Measurements above ${3.4}\,\hbox {nA}$ and ${1.7}\,\hbox {nA}$ can be made with 5 and 10% accuracy respectively. The calculation suggests that, at a bias voltage of ${0.5}\,\hbox {V}$, we can read resistance of devices up to ${100}\,\hbox {M}\Omega$ before precision starts to degrade. Overall, the instrument’s resolution and noise performance is a combination of the base performances of the key components selected for its assembly and the additional averaging performed in the FPGA. With further averaging, it may be possible to push the maximum resistance up to $\approx {1}\,\hbox {G}\Omega$, but diminishing returns will impose practical limits. The effect of changing ranging resistors is clearly visible in the figure as step discontinuities in the error magnitude.

### Pulse characteristics

**Figure 7:**
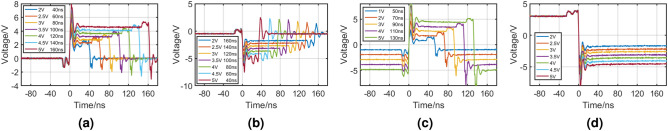
Oscilloscope captures of a variety of pulses produced with the high speed pulse generator. (**a**) +VE pulses starting at ${0}\,\hbox {V}$. (**b**) −VE pulses starting at ${-0.5}\,\hbox {V}$. (**c**) +VE pulses symmetrical around ${0}\,\hbox {V}$. (**d**) Continuous pulses starting at ${3}\,\hbox {V}$.

Here, we tested the quality of short duration pulses produced by the high speed drivers, as well as the delay mismatch between channels. We commanded a range of pulses with varying high and low values, in increments of ${10}\,\hbox {ns}$ between the minimum pulse width (${40}\,\hbox {ns}$) and ${160}\,\hbox {ns}$, and measured the output of the driver circuit. Although the instrument is capable of producing pulses with high and low states anywhere within the range of the DACs at $\pm {13.5}\,\hbox {V}$, we were only able to test pulses between $\pm {5}\,\hbox {V}$ due to limits of the high speed probes that were available. Repetition rates above ${1}\,\hbox {MHz}$ were found to cause significant heating in the driver circuits during prolonged testing, but shorter pulse trains with a repetition rate of up to ${12.5}\,\hbox {MHz}$ should be possible. The rise and fall times were all comparable, at 2–4 ns (Fig. 7). We observed a maximum mismatch of ${1.5}\,\hbox {ns}$ between channels. This is small enough to enable differential write operations (for example the biphasic pulses described in “Subsystem overview” section).

### Digital terminals

The instrument has two banks of digital channels (Table 1): a ’selector’ bank of 32 serially addressed digital outputs and an ’arbitrary level logic’ bank of 32 IO pins.

The ‘selector’ bank supports HI and LO voltages anywhere within the full $\pm {13.5}\,\hbox {V}$ range at a guaranteed minimum resolution of ${600}\,\mu \hbox {V}$. Rise times are determined by an $~{100}\,\hbox {ns}$ switch closing time plus the RC defined by the on-resistance of the switch (${9.5}\,{\Omega }$). Fall times are determined by the RC of a pull-down circuit with $R_{PD} = {8.2}\,\hbox {k}\Omega$. The circuit is configured in such way that the user can set the nominal HI voltage to be lower than LO, thereby swapping the roles of the switch and the pull-down/up resistor. This can be used, for example when a very fast fall time is required. The minimum pulse length on any pin is approx. ${1.3}\,\upmu \hbox {s}$. This is limited by the time required to write to the serial registers that control the selector states.

The ‘arbitrary level logic’ bank is a more conventional array of bidirectional level shifter ICs, with a selectable HI level of between 1.8-5.5V, at a resolution of ${120}\,\upmu \hbox {V}$. This bank is operated in parallel directly from the FPGA IO pins and as a result can operate at much higher frequency than the selector bank. The level shifters have typical rise and fall time of between ${1.3}\,\hbox {ns}$ and ${4}\,\hbox {ns}$, depending on the voltage level set. The typical propagation delay is also dependent on the selected voltage level and is typically below ${8}\,\hbox {ns}$, except at very low voltage levels, where the delay in output configuration may be as high as ${20}\,\hbox {ns}$.

**Table 1:** Selectors and arbitrary logic specifications.

	Selectors	Arbitrary logic
No. of channels	32	32
High value range	$\pm {13.5}\,\hbox {V}$	1.8–5.5 V
Low value range	$\pm {13.5}\,\hbox {V}$	${0}\,\hbox {V}$
Direction	Output	Input/Output

## Application examples

In order to illustrate the general and versatile nature of the developed instrument we have performed a set of three example tasks as shown below. First, a classical component characterisation routine was ran on a resistor, a diode, and a transistor. Second, a set of read-out operations were conducted on a crossbar array. Third, the I/O characteristics and DNL of a DAC IC were measured. This set of tasks covers a broad range of communities ranging from device development and emerging technologies to more traditional circuit design and component testing. All single component tests were conducted using a ZIF socket daughterboard as shown in Fig. 11a.

### Diode and transistor characterisation

**Figure 8:**
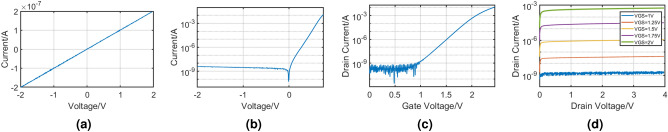
IV characteristics of a small selection of components. (**a**) IV sweep of a ${10}\,\hbox {M}\Omega$ resistor. (**b**) IV sweep of a 1N4148 diode, from ${-2}\,\hbox {V}$ to ${0.75}\,\hbox {V}$. c) Gate terminal and d) drain terminal sweeps of a 2N7000 nFET.

First, we connected a ${10}\,\hbox {M}\Omega$ resistor between two SMU channels and demonstrated IV sweep capability. One channel was configured to drive an arbitrary voltage, and the other was configured to measure current. An IV sweep between $\pm {2}\,\hbox {V}$ was conducted, with steps of ${4}\,\hbox {mV}$. Results are shown in Fig. 8a. The same test was then conducted with a 1N4148 small-signal diode (Fig. 8b). We observe that all results are above the noise floor, even in the reverse bias range. In the diode test, data points from ${0.75}\,\hbox {V}$ and up have been omitted from this figure, as the rapidly increasing forward bias current saturates the TIA and the voltage across the diode is no longer controlled.

Next, we tested a 2N7000 nFET, requiring simultaneous control of three SMU channels. First, we set its drain-source voltage $V_{DS}$ to ${1}\,\hbox {V}$ and swept the gate-source voltage $V_{GS}$ between 0-4V resulting in Fig. 8c. At below approx. 1V we hit the noise floor whilst above approx. 2.4 V we hit soft compliance as with the diode beforehand. Second, we performed a set of $V_{DS}$ sweeps between 0-4V at different $V_{GS}$ levels as shown in Fig. 8d.

### Resistive crossbar handling

The instrument is capable of controlling crossbar arrays and conducting parallel read and parallel write operations. The general read and write configurations used in the RRAM community form an illustrative and instructive set of tasks for showcasing what array-level computation frequently involves. Fig. 10 shows some examples of reading from and writing to a selectorless crossbar array (a-b), as well as interfacing an array featuring transistor-based selector devices (c-d) for either reading or writing. In all cases the array can be conceptually split into the ‘active wordline’ where bias is applied, the ‘active bitline’, from which we may choose to measure, and the inactive word- and bitlines that need to be handled appropriately for avoiding sneak path issues. In the case of the selector-based array we also need to control the selector terminals.

In general, word- and bitlines require analogue control (both for applied voltage/current and read-out) whilst the selector terminals can be used in either modes. SMU channels can be mapped to any line requiring analogue control and enables all operating options shown in Fig. 10 as well as others (e.g. where we write by raising the active wordline to $+V_{WRITE}/2$, setting the active bitline to $-V_{WRITE}/2$ and keeping all inactive lines grounded). To write, either the high speed pulse generators or the TIA can be used. Arbitrary waveforms and slower pulses can be achieved by varying the DAC+ terminal of each channel in-operando. With 64 SMU channels the system can handle up to a $32\times 32$ selectorless crossbar array, or 21x21 array with transistor selectors under analogue control. If the selectors can be satisfactorily controlled using the specialised, digital selector terminals, a $32\times 32$ array with transistor selectors is supported.

**Figure 9:**
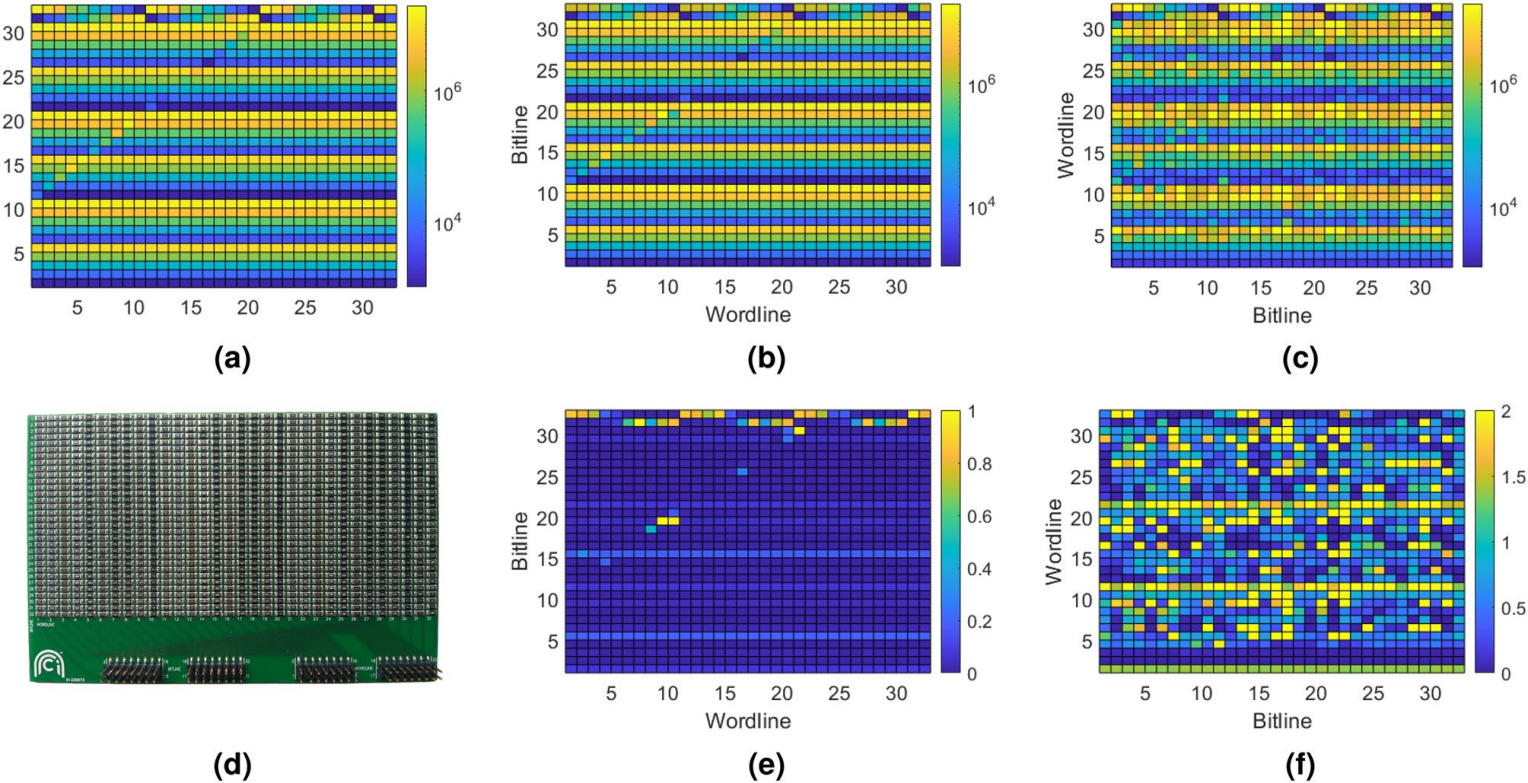
Array read operations for a $32\times 32$ resistor array. (**a**) shows the array as designed, with resistors ranging from ${1}\,\hbox {k}\Omega$ to ${15}\,\hbox {M}\Omega$. The colourbar is scaled from ${1}\,\hbox {k}\Omega$ to ${20}\,\hbox {M}\Omega$ Figure. (**b**) shows the array as read in columns. (**e**) shows the proportional error of b. (**c**) shows the array as read in rows. (**f**) shows the proportional error of (**c**).

**Figure 10:**
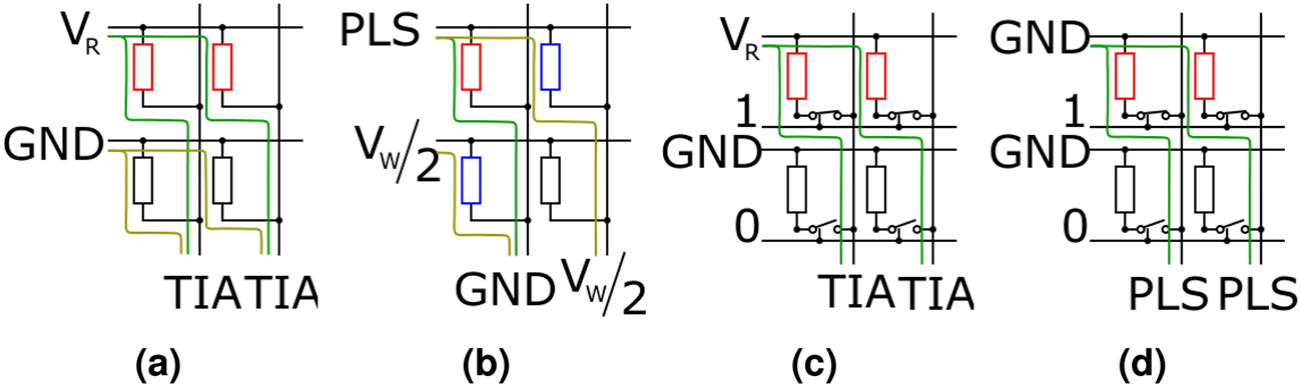
Basic read (**a**) and write (**b**) operations for selectorless crossbar arrays. (**c**) and (**d**) show the same operations for selector enabled arrays. Red, blue and black devices correspond to selected, half-selected and unselected devices. Intended current paths are shown in green, sneak paths shown in yellow.

For this work, we chose to demonstrate a read-out on a physical $32\times 32$ selectorless crossbar array of SMD resistors (Fig. 9d). The behaviour of an RRAM array can be approximated as a resistor for a single read voltage and this array provides known impedances from which read error can be calculated. The scheme used is seen in Fig. 10a: The active wordline is biased with the DC read-out voltage and the active bitlines are set to virtual grounds through the measurement set-up. For line-parallel read, all bitlines are active simultaneously. Multiple readings are taken and then averaged to improve precision (32 in our implementation). Naturally, line resistances and small errors in the DAC output voltages referencing the read-out SMU TIAs, etc. will all combine to introduce some errors through sneak paths. We sought to assess the extent of these imperfections in our subsequent measurements.

The array used 1% resistors of ${1}\,\hbox {k}\Omega$ to ${10}\,\hbox {M}\Omega$ and 5% resistors of ${15}\,\hbox {M}\Omega$; its nominal design is shown in Fig. 9a. To test read-out accuracy we simply performed a line-parallel read on each row and then calculated the fractional error $|(R_{\text {meas}}-R_{\text {actual}})/R_{\text {actual}}|$. Because the array is square we could use the same physical array to perform two separate tests: one on the array ‘as-is’ and another with the array rotated by $90^o$. This allows us to illustrate the well-known issue that the value read at any point in the array depends on the states of its neighbours^14^.

In the read operation we used, we found that bitline-to-bitline TIA reference mismatch degraded accuracy when trying to measure high-value resistors with low-value resistors on the same bitline. Even small differences in voltage between bitlines can cause non-trivial sneak currents to flow between them if both lines have a low resistance connection to an inactive wordline. The channel-to-channel voltage discrepancy is typically only ${500}\upmu \hbox {V}$, but if the ratio between the smallest device on a bitline and the device being read is comparable to the ratio between the read voltage and the mismatch voltage then accuracy will suffer. Our test used a read voltage of ${5}\,\hbox {V}$, which gives a ratio of 10000. In a configuration where the devices on a bitline are largely of the same value (Fig. 9b) the performance is excellent, with 802 of 1024 resistors measured with less than 5% error (Fig. 9e). Reading from the other direction (Fig. 9c), the ratio between the largest and smallest devices on most bitlines is 15000. In this configuration, only 171 of 1024 resistors were measured with less than 5% error and 758 measured with less than 100% error (Fig. 9f). The instrument was manually calibrated for this experiment but ADC offset was not taken into account (typ. $\pm {160}\,\upmu \hbox {V}$). As such, the channel to channel offset voltage may be higher than expected. Automated calibration will mitigate this issue. Since the resolution of a voltage read operation is greater than the DAC resolution, it should be possible to measure the channel to channel offset and use deconvolution to obtain more accurate values, but this is beyond the scope of this paper.

### Mixed signal testing

**Figure 11:**
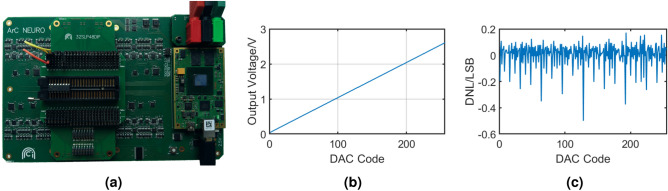
Results from an automated test of an AD558J DAC (**a**) in ${2.56}\,\hbox {V}$ range. (**b**) shows the output from code 0 to code 255. (**c**) shows the normalised differential non-linearity.

For our final test we exchanged the daughterboard used for previous test with a specialised version carrying a 48-pin ZIF socket (see Fig. 11a) and used that to test an AD558J DAC^2^. We measured the input/output transfer characteristics (digital code to analogue output) and differential non-linearity (DNL). The IC was mounted in the ZIF socket (Fig. 11a) and the board configured with jumpers to connect pins 1-8 to digital outputs and pin 11 to an onboard power supply.

Pins 9, 10, 12, 13, and 16 were connected to analogue channels and pins 14 and 15 were shorted to pin 16 with jumper wires. The analogue channels on pins 9, 10, 12, and 13 were grounded and the supply pins was set to ${10}\,\hbox {V}$. This configuration sets the IC as a $0-{2.56}\,\hbox {V}$ DAC with transparent input latches. The digital inputs were then stepped through all input codes and the voltage measured at pin 16 at each step (Fig. 11b). We observed a maximum DNL of 0.5 LSB (Fig. 11c), matching the DNL specified on the datasheet. The daughterboard used here was configured with jumpers, but a version designed around analogue matrix switches could allow for a greater degree of automation.

## Discussion and conclusions

In this paper, we have presented a general-purpose instrument that can accommodate the testing needs of a large variety of electronics component, featuring an appropriately large number of semi-independent source-metre channels. This new tool addresses an important demand for testing increasingly complex circuits while minimising the occasions where an entire PCB-based system needs to be designed to meet the requirements of the device under test. We note that important enabling factors for building such instrumentation include: (a) the availability of FPGAs with large numbers of pins (allowing high parallelism), (b) increasingly accessible multi-layer PCBs, (c) the improvement of discrete components such as amplifiers and power supplies.

Importantly, the presented instrument achieves competitive specifications to several established benchtop instruments whilst remaining in a smaller, desktop format. Table 2 summarises the achieved key performance metrics and compares them to other, established instruments in the area. Our results demonstrate how parallelism and portability can been traded against accuracy but not necessarily speed. While the low sampling rate of this system limits it to DC characterisation, the parallel structure allows for 1024 device arrays to be read in under ${50}\,\hbox {ms}$, facilitating the high throughput testing that is required by emerging technologies. Despite trading away some accuracy we are still at the point where the instrument can measure its own leakage currents, as well as set and measure all necessary noise floors (see Fig. 5). Furthermore, as demonstrated by the example applications the achieved accuracy is more than sufficient for supporting the needs of a very wide variety of electronic technologies. We thus foresee that this new tool will significantly aid the development as well as the use of emerging electron device technologies into new applications where read/write parallelism and data-level speed can be of paramount importance. We finally, acknowledge that the portability of the proposed instrument can be advantageous for a lab-at-home experience, particularly in light of the global pandemic ongoing at the time of writing this article.

In conclusion, we envisage that in the future versatile, portable instrumentation that can handle increasingly complex, non-accuracy-critical circuits will become more commonplace, accelerating and democratising research into electronic devices, components, chips etc. much the the Raspberry Pi and Arduino systems have done for embedded software research. We hope this new instrument will play a significant role in enabling this vision, as well as serve as a concrete example of such systems can be developed and what capabilities they can achieve.

**Table 2:** Comparison between this work and similar systems.

	^28^	^23^	^29^	This work
Parallel read	N	N	N	Y
Parallel write	N	N	N	Y
Channel count	2R+2W+16D	32R+32W	4R+2W	64R/W+64D
Form factor	Portable	Desktop	Benchtop	Desktop
Min. chan. current	N/A	$\pm {1}\,\hbox {nA}$	$\pm {10}\,\hbox {nA}$	$\pm {100}\,\hbox {pA}$
Max. chan. current	N/A	$\pm {5}\,\hbox {mA}$	$\pm {500}\,\hbox {mA}$	$\pm {12}\,\hbox {mA}$
Current sample rate	N/A	$50-{1000}\,\hbox {S}\,\hbox {s}^{-1}$	N/A	${833}\,\hbox {S}\,\hbox {s}^{-1}$
Voltage resolution	$166/{665}\,\upmu \hbox {V}$	$3/{24}\,\hbox {mV}$	${1}\upmu \hbox {V}$	${78}\,\upmu \hbox {V}$
Voltage sample rate	${100}\hbox {MS}\,\hbox {s}^{-1}$	${200}\,\hbox {KS}\,\hbox {s}^{-1}$	${1.25}\,\hbox {GS}\,\hbox {s}^{-1}$	${100}\,\hbox {KS}\,\hbox {s}^{-1}$
Min. pulse width	N/A	${90}\,\hbox {ns}$	${10}\,\hbox {ns}$	${40}\,\hbox {ns}$
Max. chan. current	N/A	$\pm {5}\,\hbox {mA}$	$\pm {500}\,\hbox {mA}$	$\pm {12}\,\hbox {mA}$
Pulse volt. range	$\pm {5}\,\hbox {V}$	$\pm {12}\,\hbox {V}$	$\pm {20}\,\hbox {V}$	$\pm {13.5}\,\hbox {V}$
Power	${500}\,\hbox {mW}$	${4.5}\,\hbox {W}$	${2}\,\hbox {W}$	${20}\,\hbox {W}$

## Data Availability

The datasets generated and analysed during this study are included in the supplementary information files. The Python-based software interface is available in the arc2control repository, https://github.com/arc-instruments/arc2control.
